# Oxidation of α-Pinene on the Ti-SBA-15 Catalyst Obtained Using Orange Peel Waste as Components of the Synthesis Gel

**DOI:** 10.3390/molecules30071627

**Published:** 2025-04-05

**Authors:** Jadwiga Grzeszczak, Agnieszka Wróblewska, Beata Michalkiewicz, Małgorzata Dzięcioł, Katarzyna Janda-Milczarek

**Affiliations:** 1 Department of Medical Chemistry, Pomeranian Medical University, Powstańców Wlkp. 71 St., 70-111 Szczecin, Poland; jadwiga.grzeszczak@pum.edu.pl; 2 Department of Catalytic and Sorbent Materials Engineering, Faculty of Chemical Technology and Engineering, West Pomeranian University of Technology in Szczecin, Piastów Ave. 42, 71-065 Szczecin, Poland; beata.michalkiewicz@zut.edu.pl; 3 Center for Advanced Materials and Manufacturing Process Engineering (CAMMPE), West Pomeranian University of Technology, Piastów Ave. 45, 70-311 Szczecin, Poland; 4 Department of Chemical Organic Technology and Polymer Materials, Faculty of Chemical Technology and Engineering, West Pomeranian University of Technology in Szczecin, Piastów Ave. 42, 71-065 Szczecin, Poland; malgorzata.dzieciol@zut.edu.pl; 5 Department of Biology, Parasitology and Pharmaceutical Botany, Pomeranian Medical University in Szczecin, 72 Powstańców Wielkopolskich Street, 70-111 Szczecin, Poland

**Keywords:** oxidation, alpha-pinene, Ti-SBA-15, orange peels, alpha-pinene oxide, verbenol, verbenone

## Abstract

α-Pinene is a very valuable natural raw material for organic syntheses, which is of increasing interest to scientists due to its renewability and relatively low price. This work presents the studies on the oxidation of α-pinene in the presence of two mesoporous titanium-silicate catalysts: standard Ti-SBA-15 and Ti-SBA-15 material, which was obtained by a new and green way using orange peel waste as bio-templates (Ti-SBA-15_orange peels). For the synthesis of the Ti-SBA-15 catalysts, the following raw materials were used: Pluronic P123 as the template (template usually used in the synthesis of SBA-15 materials), tetraethyl orthosilicate as the silicon source, hydrochloric acid, deionized water, and tetraisopropyl orthotitanate as the titanium source. For the synthesis of Ti-SBA-15_orange peels, a catalyst was also properly prepared, and orange peel waste as the co-templates (renewable templates) were used. The two obtained Ti-SBA-15 materials were characterized by the following instrumental methods: XRD, SEM, EDX, UV-Vis, and FTIR. Moreover, the specific surface area and pore size distribution were investigated for these catalysts with help from the nitrogen adsorption–desorption method. Catalytic tests of the obtained catalysts were performed in the oxidation of α-pinene with oxygen and by the method which did not use any solvent (α-pinene was simultaneously the raw material and solvent in this process). During the catalytic tests, the effect of temperature, catalyst content, and reaction time on the selectivities of the appropriate products and the conversion of α-pinene were studied. Depending on the conditions of the oxidation process, the catalyst obtained with the use of orange peels as co-templates showed similar or even higher activity than the standard Ti-SBA-15 catalyst.

## 1. Introduction

Industrial applications of the natural products, including the essential oils and their components, is a very promising area, where regular growth is observed, as one of the elements of a “return to nature” trend. It involves not only their use as fragrances and flavors, but also as a source of precious biologically active compounds for various applications or the substrates for the obtaining of other value-added products. The modern approach to the processes of their isolation or transformation requires following the green chemistry principles as well as development of various post-production waste disposal methods.

Sweet oranges, *Citrus sinensis* (L.) Osbeck, are one of the most widely cultivated fruit species in the world and are mainly destined for the production of orange juice. The orange peel, which is a waste product, accounts for almost 50% of the fruit’s fresh weight. It is a part rich in essential oils, simple sugars, and many valuable biologically active compounds [1,2,3,4]. Orange peels are fruit waste generated in very large quantities, mainly by the juicing industry. Their management is a major challenge, as uncontrolled disposal can adversely affect the environmental and socio-economic dimensions of sustainability. For example, their burning is associated with the release of large amounts of the greenhouse gases. These emissions have negative impact on air quality, causing asthma, lung cancer, and mortality from respiratory diseases. On the other hand, open storage of the peels requires a large land area, and their leachate can contaminate groundwater. Therefore, various strategies of disposal and recycling of this waste are necessary in order to avoid undesirable environment and health effects. In addition to being used as animal feed, orange peel waste is currently applied in a number of industries, including using as a raw material for textiles [5,6], vegan leather [7], production of biogas [8], adsorbent for wastewater treatment [9,10] and dye recovery [11]. Furthermore, they can be an additive in the production of cookies [12], a source material for the production of functional gels for the food industry [13] and a source of the essential oils [8]. They are also used in the sustainable production of amino acids by *Corynebacterium glutamicum* [14] and in the production of polyesters applied as a packaging for the food industry [15]. An interesting and recently developed direction is the use of orange peel waste for production of the catalysts of various chemical reactions [16,17,18,19].

α-Pinene is one of the monoterpenes widely distributed in nature, belonging to the group of the most important constituents of the essential oils. Natural sources of this compound are coniferous trees, *Juniperus* species, citrus fruits, and other plants; it is also one of the main components of turpentine [20]. α-Pinene is characterized by the excellent safety profile and a wide range of pharmacological activities [21], including anti-cancer [22,23], anti-inflammatory [24], antimicrobial [25], anti-allergic [26], and others. Due to the wide availability and low price, α-pinene is also a valuable raw material for the synthesis of other useful compounds, characterized by its interesting biological activity or industrial utility. For this reason, extensive research is being conducted on various processes of its transformation towards other products, including isomerization and oxidation using heterogeneous catalysts. The structure of α-pinene contains a double bond, and therefore its oxidation reaction is easy to occur. Investigation of the thermal autoxidation mechanism of α-pinene showed the complexity of this radical process, which resulted not only in the formation of α-pinene oxide but also in a variety of other products, including hydroperoxides (pinocarvyl-hydroperoxide, pinenyl-hydroperoxide, myrtenyl-hydroperoxide, and verbenyl-hydroperoxide), alcohols (pinocarvol, pinenol, myrtenol, and verbenol), aldehydes (myrtenal), and ketones (pinocarvone and verbenone) [27,28].

The example of a green approach to the oxidation process can be the application of hydrogen peroxide or oxygene in the presence of an effective catalyst. An important group of advanced catalysts which can be used for oxidation are titanium silicate zeolites [29]. They belong to the group of mesoporous catalysts and can be generally synthetized using two methods: (1) direct hydrothermal synthesis in which a titanium precursor (e.g., tetrabuthyl orthotitanate, TiCl_4_, or Ti(OiPr)_4_) is added together with a silica precursor (most often tetraethoxysilicon) to the crystallization gel (co-condensation of Si and Ti sources) and (2) by post-synthesis titanation with the appropriate titanium source, in which Ti is introduced into the already formed SBA-15 structure by reaction with silanol groups on the surface [30]. The studies on various modifications of the methods for obtaining these catalysts in order to increase their catalytic activity are described in the literature [31,32,33,34,35,36,37]. These modifications are mainly aimed at increasing the durability of the Ti-SBA-15 or SBA-15 structure (in the case of post-synthesis titanation as the method of obtaining Ti-SBA-15), as well as increasing the accessibility of active centers associated with titanium, on which catalytic reactions involving hydrogen peroxide or oxygen take place in the Ti-SBA-15 structure and increasing the hydrophobicity of the obtained catalyst by, for example, silylation [32,33,35,37]. The active centers of Ti must be well dispersed in the structure (centers in the form of Ti^4+^ incorporated into the silica structure, the presence of Ti–O–Si bonds in the structure), while the amount of titanium present in the form of anatase should be limited because this compound blocks the pores of the catalyst and reduces its activity. In the case of the co-precipitation method, it is important to select the appropriate Ti precursor, such a precursor should not hydrolyze too quickly, which prevents the formation of anatase, and at the same time it should mix well with tetraethyl o-silicate, so that titanium is incorporated into the silica structure. In order to obtain well-dispersed active titanium centers, it is also important to maintain a low concentration of the Ti precursor—usually below 5 mol%—and a lower pH (slightly acidic conditions), which reduces the rate of hydrolysis and condensation of the titanium precursor. A lower temperature of obtaining the crystallization gel reduces titanium aggregation and the formation of anatase, and the slow addition of the Ti precursor allows for better integration with the SiO_2_ network [31,38,39]. In the case of the method in which the SBA-15 material is obtained first, and then titanation is carried out, the conditions for obtaining the SBA-15 material are also important, as they significantly affect the final activity of the material. Of great importance here are such parameters as follows: hydrothermal aging temperature [32], but also, for example, an appropriate amount of organic ammonium hydroxide [36]. Studies by P. Wu and T. Tasumi [36] have shown, for example, that during the synthesis of Ti-SBA-15 by titanation of pure silica SBA-15 material in glycerol and in the presence of quaternary organic ammonium hydroxides, an appropriate, elevated temperature is essential, and the nature of Ti spaces depends on the Si/Ti ratio. At Si/Ti ratios higher than 50, mainly Ti species were obtained in the form of tetrahydrally coordinated Ti in isolated states (the Ti form required for the catalytic activity of Ti-SBA-15), while poorly dispersed Ti species of octahedral coordination were formed at higher Ti incorporation levels (unfavorable Ti form). Moreover, M. Kosmulski and E. Maczka [34] described metal-doped silcas (these were materials that, among others, contained Ti), in which the properties of the materials obtained by this method depended on the time and temperature of deposition and proportion of the reagents. An important process during the preparation of the Ti-SBA-15 catalyst is also the calcination stage, during which very high temperatures should be avoided because a too high calcination temperature (above 600 °C) can lead to the formation of anatase or to the destruction of the porous structure of SBA-15 or Ti-SBA-15. Calcination is usually carried out at a temperature of 500–550 °C in an air atmosphere, with a controlled heating rate [32,39].

The subject of the green synthesis of mesoporous materials, including Ti-SBA-15 [40] type catalysts based on the use of plant extracts, individual substances of natural origin, or selected parts of plants (co-templating agents) in the form of components added to the crystallization gel, is new, and there are few literature reports on this subject. This green approach to the synthesis of these porous materials, by using substances of plant origin (plant-extract-assisted synthesis or bio-templating), is based on the assumption that the natural component of the crystallization gel can have a beneficial effect on the porous structure and surface properties of the obtained material. In the scientific literature, however, there are more reports on the green synthesis of materials related to mesoporous silicas, for example, TiO_2_ nanoparticles. In the case of obtaining TiO_2_ nanoparticles, for example, the use of an aloe leaf extract has been described. Using this extract, nanoparticles with high photocatalytic activity were obtained, and they can also be used in other applications, such as hydrogen production [40,41]. G. Nabi et al. [42] described the use of Citrus Limetta extract for obtaining spherical TiO_2_ with excellent photocatalytic activity (almost 90% dye was degraded within 80 min). The citric acid present within the extract acted as a reducing and capping agent for nanoparticles, resulting pure TiO_2_ nanoparticles. The synthesized nanoparticles were approximately 80–100 nm in size and spherical in shape, and the distribution of the nanoparticles was almost uniform.

The scientific literature also describes the use of selected parts of appropriate plants as components of crystallization gels used as templates. An example is the synthesis of mesoporous TiO_2_/SiO_2_ composite with aquatic plant leaves from reed, water hyacinth, and duckweed described by Duan et al. [43]. During the performed studies, the TiO_2_/SiO_2_ samples templated by reed and water hyacinth leaves exhibited high photocatalytic activity, while the TiO_2_/SiO_2_ samples obtained from duckweed were less active in the degradation of gentian violet. R. Wu et al. [44] described the preparation of TiO_2_/P,K-containing grapefruit peel, biochar composites with enhanced photocatalytic activity. Grapefruit peel powder was used in the synthesis of these materials. The analysis of the results indicated that the grapefruit peels played a significant role in the formation of the porous architecture of the TiO_2_/P, K-PC composites. M. M Abomughaid [45] described the synthesis of silica nanomaterials from orange peels. Silica nanoparticles were synthesized using the aqueous extract of orange peels. During the studies, silica nanoparticles with a spherical and amorphous nature and an average size of 20 nm were obtained. The synthesized silica nanoparticles were highly stable, pure, and spherical in nature. K. Zanotti et al. [46] described the synthesis, through the sol-gel technique, of silica-based materials using citrus bio-waste; it was the first stage of the development of new silica-based supports that can be used in heterogeneous catalysis. This method was carried out by the acid or alkali hydrolysis combined with bio-waste, such as orange and lemon peels. The main goal of these studies was to obtain silica-based materials starting from tetraethyl o-silicate as a precursor and using different acids, such as acetic, citric, and hydrochloric acid and ammonium hydroxide, and also by adding different amounts of lemon and orange peels. The research was carried out in order to find the influence of bio-waste on acids/alkali-precursor hydrolysis and also to find the possibility of the replacement of these acids by orange or lemon peels in the future. The studies showed that the bio-waste used can provide acidity to partially replace the acids usually used in the formation of crystallization gels for acid hydrolysis of a Si source (the values obtained for tests with 25% and 50% of bio-waste were similar to the test without bio-wastes), but it was not possible to replace them completely. In addition, the materials obtained with lemon and orange peels also showed the ability to maintain the siliceous structure.

M. Brigante and M. Avena [47] described the synthesis of mesoporous silica materials in acidic media, using tetraethyl orthosilicate as a silica precursor and with the water-soluble biopolymer hydroethyl starch as a template (hydroxyethyl starch is a non-ionic starch derivate). They studied the effect of the synthesis temperature on the morphology and pore structure of the obtained materials. The results showed that, by a changing of the temperature, the morphology of the obtained materials was changed from slices with worm-like mesopores of around 5 nm to microporous lamellas when the temperature synthesis was increased from 60 °C to 100 °C, respectively. These changes were attributed to a partial degradation of the template that hindered the gel formation and disrupted its interaction with the silica species through H-bond formations and/or electrostatic interactions.

U. Sultan et al. [48] described studies using bio-based limonene as an alternative expander molecule for the synthesis of large-pore templated silica (SBA-15) instead of the commonly used 1,3,5-trimethylbenzene (TMB). The conducted TEM and SEM analyses indicated similar pore and particle morphologies of silicas obtained with limonene and TMB, which shows that limonene can be a good substitute for fossil-based TMB because it can fully overtake its function as a pore expander.

The process of allylic oxidation of α-pinene using aqueous H_2_O_2_ was studied in various conditions over different catalysts, among which H_5_PW_11_TiO_40_/silica and Ti-MCF were found to be the most effective and lead to a verbenol/verbenone mixture [39]. Oxidation of α-pinene with hydrogen peroxide using Ti-MCM-41 heterogeneous catalysts resulted in obtaining verbenol, verbenone, and campholenic aldehyde as the main products [38]. The application of ZSM-5 catalysts enabled green, solvent-free oxidation of α-pinene with oxygen, preferable to verbenone, verbenol, and α-pinene oxide [49]. The solvent-free α-pinene oxidation processes were also conducted using TS-1 catalyst [50], FeCl_3_-modified carbonaceous catalysts from orange peels [18], and clinoptilolite [49].

The aim of our research was to carry out the oxidation process of α-pinene over two Ti-SBA-15 catalysts, where one of them was a standardly obtained material (standard Ti-SBA-15), while the synthesis of the second Ti-SBA-15 catalyst was carried out with the addition of biomass waste to the crystallized gel in the form of suitably prepared orange peels, which constituted co-templates (the second catalyst was named as Ti-SBA-15_orange peels). Such an unconventional use of orange peels as templates could in the future reduce the costs of the synthesis of zeolites and zeolite-like materials, if the standard, often very toxic templates (ammonium compounds), were at least partially replaced with templates of natural origin. During the studies on the oxidation process, the influence of the temperature, the catalyst content, and the reaction time of α-pinene conversion and the selectivities of the appropriate products were analyzed. The results obtained for both catalysts were compared to answer the question of whether the use of biomass waste for the synthesis of mesoporous Ti-SBA-15 material allows for the production of a comparably active or perhaps even more active catalyst in comparison to the standardly obtained Ti-SBA-15 catalyst. It should be emphasized that the presented method of the obtaining of the Ti-SBA-15_orange peel catalysts and carrying out the α-pinene oxidation process in its presence has not been described in the literature so far. This process is in line with the actual trends in green chemistry due to the application of natural, renewable raw materials for production of other valuable products via environmentally friendly methods. In addition, it pursues the goals of a circular economy that aims to utilize any waste fractions, pointing out the possibilities of unconventional use of problematic bio-waste—in this case, orange peels. The main products obtained during the studied process were the following: α-pinene oxide, verbenone, and verbenol. These products are very valuable compounds applied as additives in food, beverages, perfumes, and cosmetics. Also, by-products of the process of oxidation of α-pinene, which were detected in the post-reaction mixtures (pinocarveol, myrtenal, myrtenol, carveol, carvone, and pinanediol), have a lot of practical applications.

## 2. Results and Discussion

### 2.1. Characteristics of Ti-SBA-15 Materials

The results of the instrumental studies presented below were aimed at checking the effect of using the bio-template in the form of dried and crushed orange peels on the morphology of the mesoporous structure of the titanium-silicate material Ti-SBA-15 formed in the presence of this bio-template (including the size of the mesoporous silica particles, their shape, and the pore size), as well as on the number of active centers in the form of titanium (Ti^4+^) tetrahedrally coordinated in the silica structure and on the inhibition of anatase formation (titanium oxide, which blocks the catalyst pores, reducing its activity, while by binding Ti in this oxide, the amount of Ti tetrahedrally bound in the silica structure, which is the active center of this catalyst, is reduced, which also leads to a reduction in the catalyst activity).

**Figure 1:**
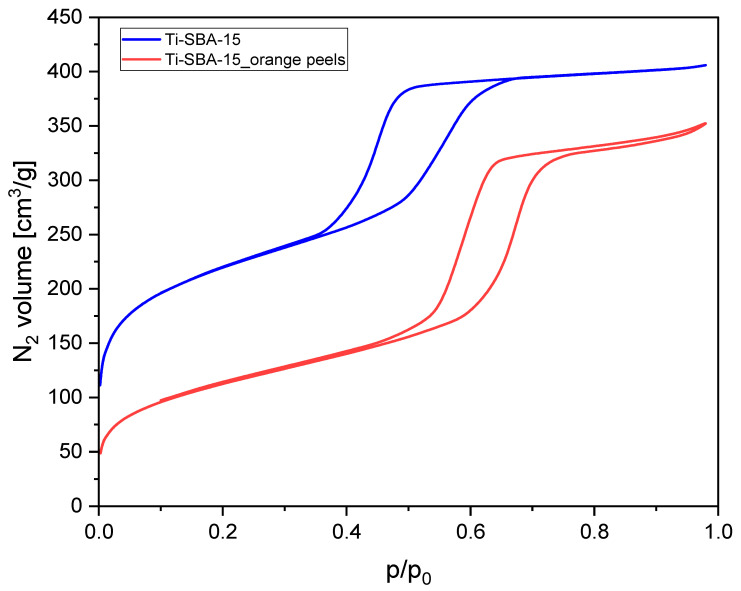
Nitrogen adsorption–desorption isotherms of the Ti-SBA-15 materials (standard Ti-SBA-15 and Ti-SBA-15_orange peels (material synthesized with the addition of ground orange peels)).

Figure 1 shows nitrogen adsorption–desorption isotherms measured at 77 K for Ti-SBA-15 and Ti-SBA-15_orange peel materials (synthesized with the addition of dried and ground orange peels).

Both isotherms exhibit typical Type IV behavior according to the IUPAC classification, indicating the presence of mesoporous structures [51]. The hysteresis loops in both cases are characteristic of capillary condensation within mesopores. The adsorption volume for Ti-SBA-15 is significantly higher compared to Ti-SBA-15_orange peels. This suggests that the standard Ti-SBA-15 has a higher surface area and pore volume. The hysteresis loop for both materials is of the H1 type, indicating uniform cylindrical mesopores. However, the hysteresis loop for Ti-SBA-15_orange peels is narrower, which could suggest reduced pore size or connectivity. The initial steep rise at low relative pressure is more pronounced for Ti-SBA-15, indicating a larger contribution of microporosity or higher surface area in Ti-SBA-15. The reduced adsorption volume in the red isotherm implies that the addition of orange peels during synthesis affects the mesopore formation, likely by introducing structural defects by the influence of bio-templates on the process of micelle formation and their structure.

Table 1 presents the textural properties of Ti-SBA-15 and Ti-SBA-15_orange peels.

Table 1 confirms the conclusion drawn based on Figure 1.

**Figure 2:**
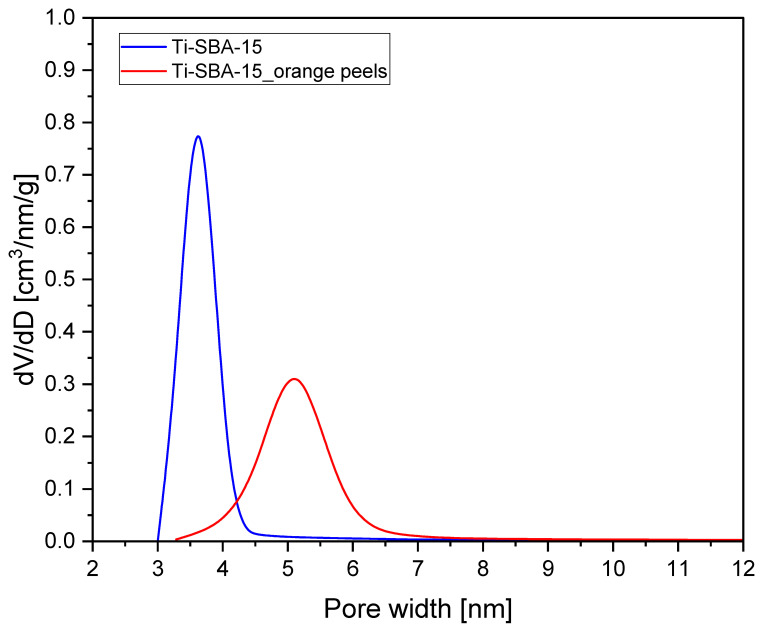
Pore size distribution of the Ti-SBA-15 materials (standard Ti-SBA-15 and Ti-SBA-15_orange peels (material synthesized with the addition of ground orange peels)).

Figure 2 presents the pore size distributions of Ti-SBA-15 and Ti-SBA-15_orange peels, determined from nitrogen sorption isotherms at 77 K using BJH method.

For Ti-SBA-15, the pore size distribution is narrow, exhibiting a sharp peak between 3.0 and 4.3 nm, with a central value of 3.6 nm. This indicates a highly uniform mesoporous structure, characteristic of standard SBA-15 materials. The sharp peak suggests well-controlled synthesis conditions and minimal structural defects. For Ti-SBA-15_orange peels, pore size distribution is broader and shifted towards larger pore widths, with a peak between 3.3 and 7 nm, with a central value of 5 nm. The broader distribution suggests less uniformity in the pore structure compared to the standard material. The shift to larger pore sizes implies that the incorporation of orange peels during synthesis affected the templating process, possibly due to changes in the micelle structure of the surfactant. The differences in pore structure are likely due to the presence of organic compounds from the orange peels in the crystallization gel, which may have altered the micelle formation or pore templating process during synthesis. Overall, the use of orange peels as an additive introduces tunability to the pore structure, but at the cost of uniformity. This trade-off can be tailored depending on the intended application of the material. More information on this subject can be found in the discussion of the SEM images. It is worth it to mention that, due to narrower pores, slower diffusion of α-pinene can be expected in the pores of Ti-SBA-15 compared to diffusion in the wider pores of Ti-SBA-15_orange peels. Higher activity of this catalyst in the oxidation of α-pinene can be expected due to the larger pore size. In catalysts with narrower pores, the dominant regime is typically diffusion-controlled, meaning that mass transport limitations play a significant role. The restricted pore diameter hinders the free movement of reactant and product molecules, especially in the case of larger substrates such as α-pinene. Internal diffusion within the channels can be significantly slowed down, making it more difficult for reactants to reach the active sites. As a result, the overall reaction rate is not determined solely by the intrinsic catalytic activity of the active centers, but rather by how efficiently the reactants can diffuse through the porous structure, i.e., the rate is limited by diffusion resistance.

In contrast, in catalysts with wider pores, the process is generally governed by kinetic control, where the rate of the chemical reaction itself becomes the determining factor. The larger pore diameter facilitates easier access of reactants to the active sites and allows for rapid removal of the products. Under these conditions, diffusion limitations are minimized, and the chemical nature and reactivity of the active sites become the primary factors influencing the overall reaction rate, rather than mass transport phenomena.

**Figure 3:**
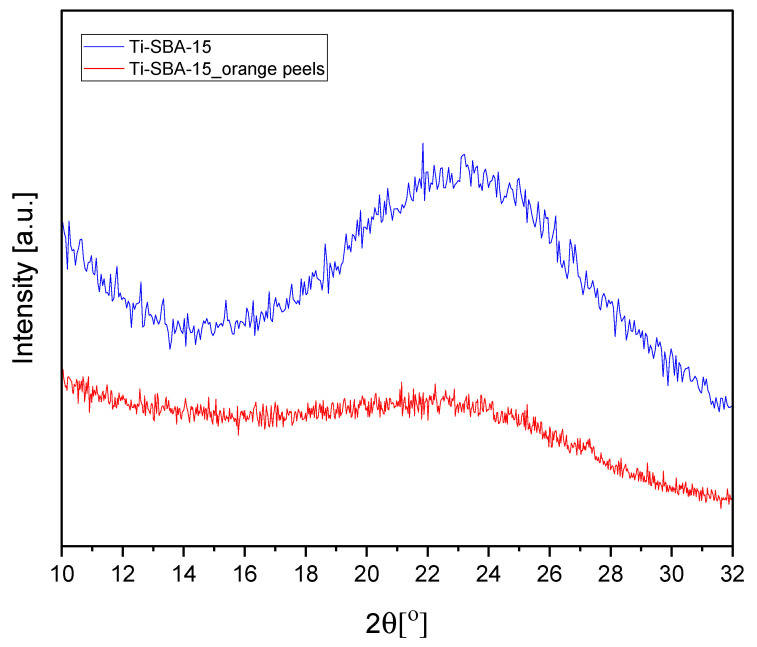
XRD pattern of the Ti-SBA-15 materials (standard Ti-SBA-15 and Ti-SBA-15_orange peels (material synthesized with the addition of ground orange peels)).

Figure 3 presents the XRD pattern of the obtained Ti-SBA-15 materials.

Both patterns exhibit broad diffraction peaks in the range of approximately 18–28° of 2θ angle, indicating the presence of TiO_2_. The intensity of the peaks for Ti-SBA-15 is significantly higher than for Ti-SBA-15_orange peels. This suggests that the standard material has a more well-defined or ordered structure compared to the material synthesized with orange peels. No sharp diffraction peaks corresponding to crystalline titanium oxide phases (e.g., anatase and rutile) are observed in either pattern. This suggests that titanium is well-dispersed in the silica matrix or exists in an amorphous state.

**Figure 4:**
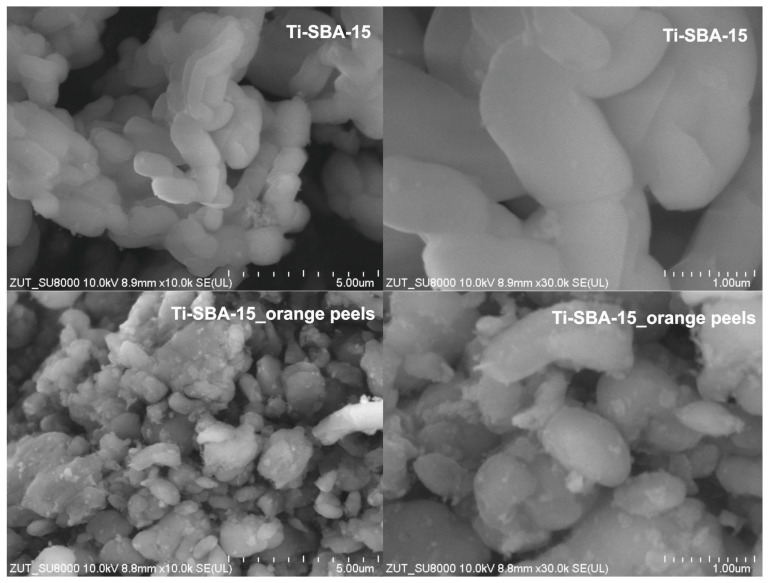
SEM pictures of the Ti-SBA-15 materials (standard Ti-SBA-15 and Ti-SBA-15_orange peels (material synthesized with the addition of ground orange peels)).

Figure 4 presents SEM pictures of the obtained Ti-SBA-15 materials.

The provided SEM images (Figure 4) show the morphological differences between Ti-SBA-15 synthesized using the standard method and Ti-SBA-15 synthesized with the addition of orange peels. The images of Ti-SBA-15 reveal the characteristic rod-like morphology of SBA-15 materials, typical of mesoporous silica synthesized using a surfactant template. The particles exhibit a uniform structure with smooth surfaces, indicative of a well-ordered synthesis. Higher magnification (30,000×) shows tightly packed and elongated particles, confirming the structural integrity and consistency of the material.

The morphology of Ti-SBA-15_orange peels is significantly altered compared to the standard material. The particles appear less uniform, with irregular shapes and rougher surfaces. Aggregation of smaller particles is observed, suggesting that the addition of orange peels disrupted the templating process during synthesis. The rough and heterogeneous appearance of the particles may result from the presence of organic compounds introduced by the orange peels, influencing the particle growth and aggregation. The rougher surfaces and irregular particle shapes in the modified material may increase surface heterogeneity, potentially enhancing interactions with certain adsorbates or reactants. This modification may offer benefits for specific applications requiring diverse surface properties.

**Figure 5:**
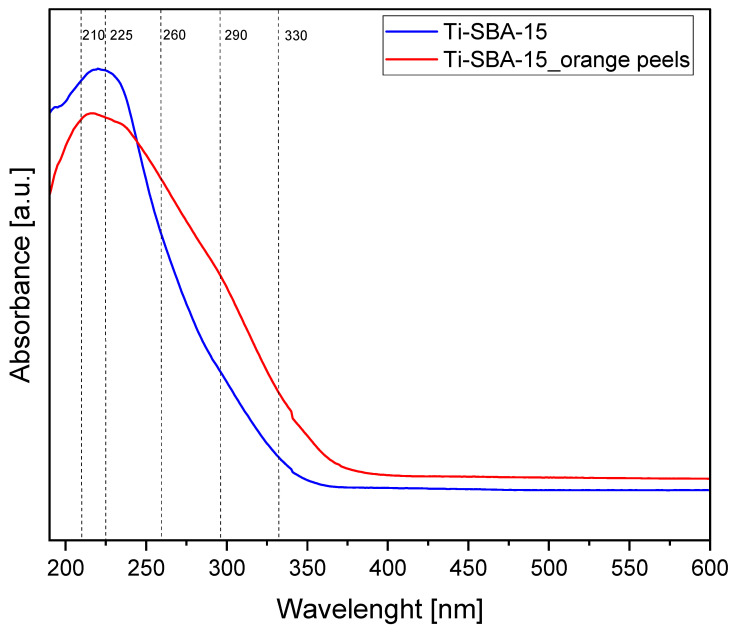
UV-Vis spectra of the Ti-SBA-15 materials (standard Ti-SBA-15 and Ti-SBA-15_orange peels (material synthesized with the addition of ground orange peels)).

Figure 5 shows the UV-Vis spectra of Ti-SBA-15 materials. The spectra show a characteristic absorption band around 210–225 nm, which indicates the presence of titanium in tetrahedral coordination in the silica structure. In addition, the spectra show bands at about 260 nm and 290 nm, which can be attributed to titanium in octahedral coordination. The change in the coordination number of titanium from 4 to 5 and 6 occurs due to the coordination of the first and then the second water molecules, which is the reason for the appearance of bands in the range of 260 and 290 nm. In addition, a small band can be observed on the spectra around 330 nm, indicating the presence of TiO_2_ in the form of anatase [52,53].

The comparison of UV-Vis spectra obtained for the standard Ti-SBA-15 material and Ti-SBA-15_orange peel material shows that although both catalyst samples contained the same amount of titanium (results presented earlier in Table 1); in the Ti-SBA-15_orange peel material sample, there was a decrease in the amount of titanium in tetrahedral coordination (Ti^4+^), which is bound in the silica structure and is the active center of the catalyst and on which the oxidation reactions of α-pinene molecules take place (band around 210–225 nm). At the same time, for this sample we can also observe an increase in the amount of titanium with coordination number 5 and 6 (bands at about 260 nm and 290 nm), which indicates that the tendency for the coordinated bonding of water molecules with titanium active centers in the silica structure increased. Coordinate bonding of water molecules with titanium active centers may make it difficult for the oxidation substrate molecules to bind to the active centers and, as a result, reduce the rate of the oxidation reaction. Moreover, for the Ti-SBA-15_orange peel material sample, an increase in the intensity of the band around 330 nm is visible, which indicates an increase in the amount of anatase in this sample compared to the standard Ti-SBA-15 material sample. This increase in the amount of anatase may also be the reason for the decrease in the catalyst activity, due to the possibility of blocking the pores by the anatase deposition in them. On the other hand, taking into account the significant increase in the pore size observed for the Ti-SBA-15_orange peel sample (Figure 2), the presence of anatase in the pores may not affect the catalyst activity. To sum up, it can be said that the use of bio-templates in the form of orange peels resulted in a small decrease in the amount of titanium incorporated into the silica structure (probably due to the lower stabilization of Ti^4+^ ions during the formation of the mesoporous structure) and an increase in the amount of titanium precipitating in the pores in the form of anatase, while maintaining the same total amount of titanium in both catalyst samples. Additionally, the UV-VIS tests showed that the titanium present in the active centers of the Ti-SBA-15_orange peel catalyst is surrounded by water molecules to a greater extent—changing its coordination number—than in the case of standard Ti-SBA-15.

**Figure 6:**
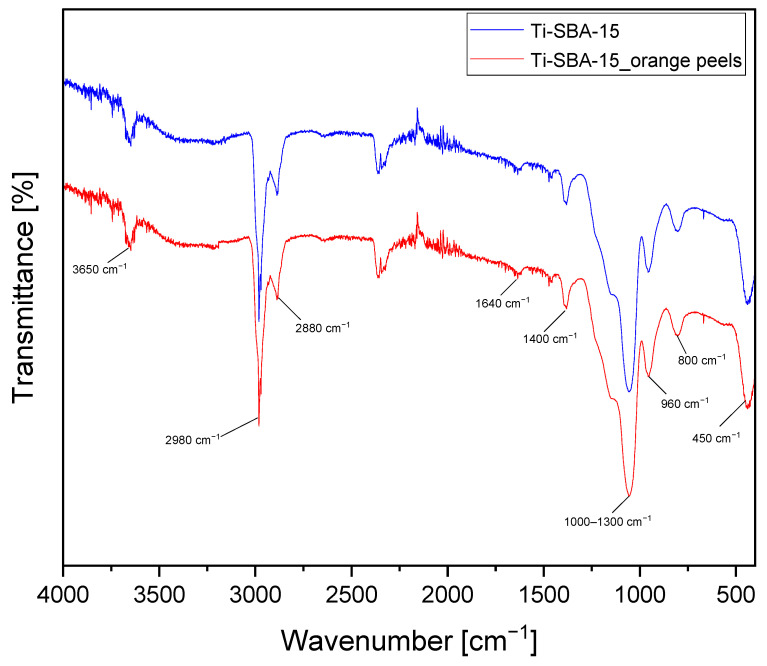
FTIR spectra of the Ti-SBA-15 materials (standard Ti-SBA-15 and Ti-SBA-15_orange peels (material synthesized with the addition of ground orange peels)).

Figure 6 shows the FTIR spectra of Ti-SBA-15 materials. The following main bands are observed in these spectra: 3650, 2980, 2880, 1640, 1400, 1000–1300, 960, 800, and 450 cm^−1^. The bands between 2880 and 3650 cm^−1^ are attributed to OH stretching vibrations of surface hydroxyls bound to silicon (Si-OH). The bands in the 1640 cm^−1^ and 1400 cm^−1^ range are attributed to bending vibrations of the -OH groups, originating from water molecules adsorbed on the surface of the material. The bands in the range of 450 and 800 cm^−1^ are attributed to bending deformations of Si-O-Si groups, in which the angle between bonds changes, and symmetric valence vibrations associated with changes in the length of Si-O-Si bonds. The band in the range of 1000–1300 cm^−1^ is attributed to the presence of Si-O-Si bonds associated with the formation of silica in the structure of the material. For Ti-SBA-15 materials, the most characteristic band is at 960 cm^−1^, which is associated with isomorphous substitution of Si by Ti ions. This band is attributed to the stress of polar Si-O-Ti bonds or the presence of a Ti = O titanyl group, which confirms the incorporation of titanium into the silica structure [52,53].

The comparison of FTIR spectra obtained for the standard Ti-SBA-15 material and Ti-SBA-15_orange peel material did not show any significant differences in the intensities of the above-mentioned main bands.

The conducted studies have shown that orange peels used as the bio-template in the synthesis of the mesoporous Ti-SBA-15 catalyst had a very large effect on the morphology of this catalyst and on the incorporation of titanium into the silica structure and thus the creation of active centers on which oxidation reactions of organic molecules occur. In comparison to the standard Ti-SBA-15 catalyst, for Ti-SBA-15_orange peel material the following were observed: the reduced adsorption volume, smaller specific surface area, the same total titanium content, larger pore sizes, less uniform particle with irregular shapes, and rougher surfaces having a tendency for aggregation and a larger amount of titanium precipitating in the form of anatase. All these differences in the morphology of the Ti-SBA-15_orange peel catalyst and in the number of active titanium sites present in its structure were most likely caused by the presence of a large number of different organic compounds in the orange peels, which influenced the structure of the formed micelles, as well as the particle growth and their aggregation. According to literature data [1,54], the chemical composition of orange peels is very complex. The main components of orange peels are the following: soluble sugars; starches; fibers, including cellulose, hemicellulose, lignin, pectin; and proteins. Important components of orange peels are also organic acids such as citric acid, malic acid, malonic acid, and oxalic acid, and also vitamins such as Vitamin C (ascorbic acid). In orange peels are present sugars such as glucose, fructose, and sucrose. Moreover, their cell walls contain insoluble polysaccharides, such as cellulose, hemicellulose, and pectin. Additionally, orange peel essential oils contain mainly the monoterpene compound—limonene. For example, during our studies on obtaining limonene from orange peels by hydrodistillation of 236.6 g of fresh orange peels (the oranges were purchased at a local supermarket), we obtained 2.34 g of orange oil containing mainly limonene with a purity of about 97% (purity was determined by gas chromatography).

It can be assumed that, among the above-mentioned organic compounds, limonene molecules may be of great importance for the morphology and catalytic activity of the Ti-SBA-15_orange peel catalyst obtained by us. In the publication by U. Sultan et al. [48], the role of limonene as a pore expander in the synthesis of mesoporous silica of the SBA-15 type was described. In the case of the Ti-SBA-15_orange peel catalyst obtained by us in this work, the effect of increasing the pore diameter was also observed in comparison to the standard Ti-SBA-15 catalyst. Taking into account the above-mentioned studies by U. Sultan et al. [48], it can be assumed that limonene molecules could have played a major role in increasing the pore diameter. Of the other compounds mentioned above present in orange peels, pectin, cellulose, hemicellulose, or starch can act as biodegradable templates, taking part in the pore formation process. Such the action of a starch derivative (hydroethyl starch) was described by M. Brigante and M. Avena [47]. In the studies of these authors, the addition of hydroethyl starch during the preparation of mesoporous silica caused changes in the morphology and pore structure, which was associated with partial degradation of the template that hindered the gel formation and disrupted its interaction with the silica species through H-bonds formations and/or electrostatic interactions. Taking into account the changes in the pore size and the shape and size of the particles of the Ti-SBA-15_orange peel catalyst obtained by us, pectin, cellulose, hemicellulose and starch, present in orange peels, could interfere in a similar way to hydroethyl starch with the process of crystallization during gel formation, as well as the process of nucleation and the formation of particles of the mesoporous Ti-SBA-15 material during our synthesis of this material. Pectin, cellulose, hemicellulose, and starch can also affect the density of the crystallization gel formed, which can also be important for the process of the hydrolysis of compounds that are the source of titanium and silicon, and therefore also affect the rate of titanium incorporation into the silica structure. However, at the current stage of our research it is difficult to describe precisely the influence of individual organic compounds present in orange peels on the structure and catalytic activity of the Ti-SBA-15_orange peel catalyst. This requires much more complicated studies, also taking into account, for example, the synergistic effect of some compounds.

### 2.2. Studies on the Catalytic Activity of the Ti-SBA_15 Materials in the Oxidation of Alpha-Pinene with Oxygen

**Figure 7:**
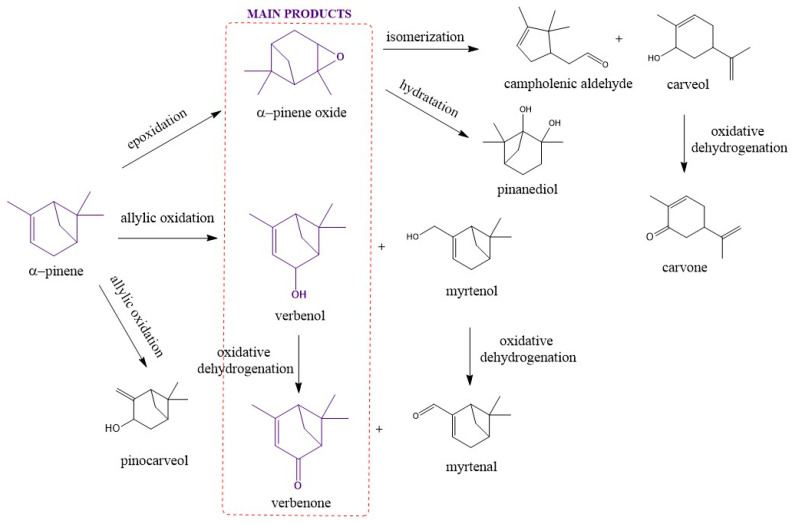
The most important reactions occurring during the catalytic studies on the oxidation of α-pinene and the main and by-products formed in this process.

The two Ti-SBA-15 porous materials obtained in this work, standard Ti-SBA-15 and Ti-SBA-15_orange peels (material synthesized with the addition of ground orange peels), were used as the catalysts in the process of oxidation of α-pinene. The catalytic tests consisted of three consecutive stages aimed at identifying the most favorable process parameters, such as temperature, catalyst content, and reaction time. The most beneficial parameters of the oxidation process conducted were determined based on the values of the selectivity of the transformation of α-pinene into the main products, such as α-pinene oxide, verbenol, and verbenone, as well as on the basis of values of the conversion of α-pinene. In the first stage, the effect of temperature was always studied at given constant values of the other two process parameters (catalyst content and reaction time). After selecting the most favorable temperature, studies were conducted on the effect of the amount of catalyst at this temperature and at the reaction time previously assumed (during the temperature effect studies). After determining the most favorable catalyst content, studies were conducted on the effect of the reaction time at the above-mentioned previously determined: the most favorable temperature and the most favorable catalyst content. Below, in Figure 7, the most important reactions occurring during catalytic studies on the oxidation of α-pinene and the main and by-products formed in this process are presented. The presence of organic acids, including citric acid, in orange peels is also of great importance for the morphology and activity of the obtained Ti-SBA-15_ orange peel catalyst, which was described, among others, in the publication by K. Zanotti et al. [46]. The presence of these organic acids affects the pH of the crystallization gel and therefore may also affect the rate of the hydrolysis of compounds that are a source of silicon and titanium. The mismatch of the hydrolysis rate of these compounds may disturb both the process of creating a mesoporous structure and the incorporation of titanium into it and the creation of active centers, through increased formation of anatase, which precipitates in the pores and blocks them.

At the first stage, the effect of temperature on the selectivities of the appropriate products and the conversion of α-pinene was studied for the standard Ti-SBA-15 catalyst. The influence of temperature was tested in the range of 80–130 °C; moreover, the catalyst amount amounted to 0.5 wt%, and the reaction time was 1 h.

**Figure 8:**
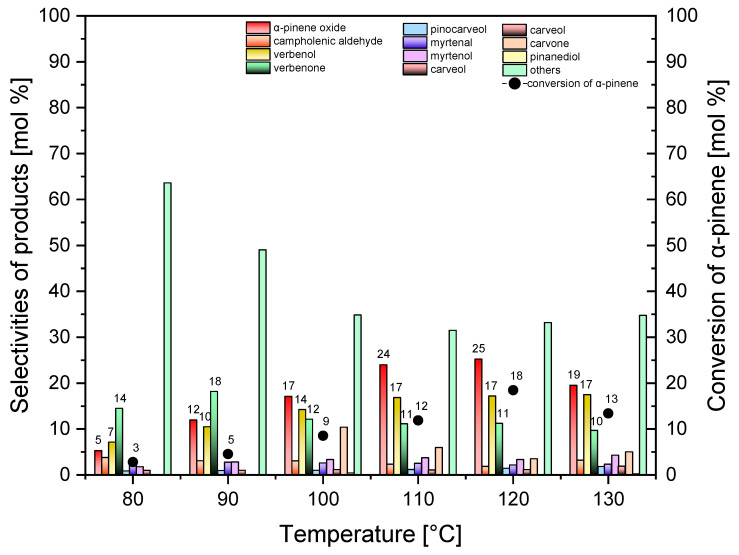
Influence of temperature on the conversion of α-pinene and selectivities of products in the oxidation of α-pinene over standard Ti-SBA-15 (catalyst content, 0.5 wt%; reaction time, 1 h). “Others” refers to the sum of the selectivities of by-products of the α-pinene oxidation process that were formed in smaller amounts.

The results of the first stage of the catalytic tests for the standard Ti-SBA-15 are presented in Figure 8.

It results from Figure 8 that, with increases in the temperature values from 80 °C to 120 °C, the selectivity of the transformation to α-pinene oxide increases from 5 mol% to 25 mol% and then decreases to 19 mol% at the temperature of 130 °C. It is also visible that, with the increase in the temperature from 80 °C to 110 °C, the selectivity of the formation to verbenol increases from 7 mol% to 17 mol%, and the next remains at this level up to the temperature of 130 °C. The increase in the values of temperature from 80 °C to 90 °C also causes the increase in the values of the selectivity of the transformation to verbenone from 14 mol% to 18 mol%, and then this function decreases to 10 mol% (for the temperature of 130 °C). In the range of temperatures from 80 °C to 120 °C, the conversion of α-pinene increases from 3 mol% to 18 mol% and then slightly decreases to 13 mol% at the temperature of 130 °C. The analysis of the results presented in Figure 8 shows that the highest value of the selectivity of the main products (alpha-pinene oxide and verbenol) and the highest conversion of α-pinene were achieved when the reaction was carried out at 120 °C. Therefore, this temperature was considered as the most beneficial and selected for the next stages of the studies.

**Figure 9:**
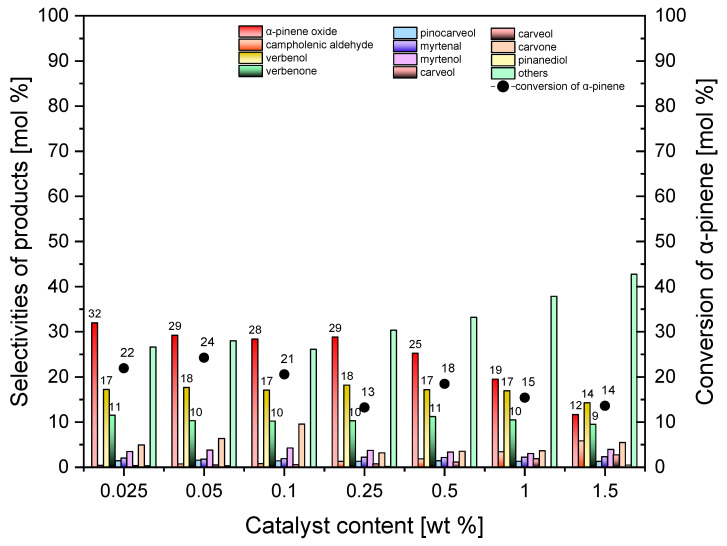
Influence of the catalyst content on the conversion of α-pinene and selectivities of products in the oxidation of α-pinene over standard Ti-SBA-15 catalyst (temperature, 120 °C; reaction time, 1 h). “Others” refers to the sum of the selectivities of by-products of the α-pinene oxidation process that were formed in smaller amounts.

The studies of the influence of catalyst content (standard Ti-SBA-15) on the selectivities of transformation to appropriate products and on the conversion of α-pinene are presented in Figure 9.

Figure 9 shows that, as the amount of standard Ti-SBA catalyst increases from 0.025 wt% to 1.5 wt%, the selectivity of the transformation to α-pinene oxide decreases from 32 mol% to 12 mol%. Moreover, in the catalyst content range from 0.025 wt% to 1 wt%, the selectivity of the transformation to verbenol remains on the level of about 17 mol%, while at the catalyst content of 1.5 wt%, the selectivity of this compound is slightly lower and amounts to 14 mol%. The selectivity of the transformation to verbenone has values in the range of 9–11 mol% in the studied catalyst content range. As the catalyst content increases from 0.025 wt% to 0.05 wt%, the conversion of α-pinene increases from 22 mol% to 24 mol%, and then this function decreases to 14 mol% for the catalyst content of 1.5 wt%. Taking into account mainly the values of the conversion α-pinene, and in the second place the selectivities of the main 3 products, it was taken that the most beneficial catalyst content is 0.05 wt%.

**Figure 10:**
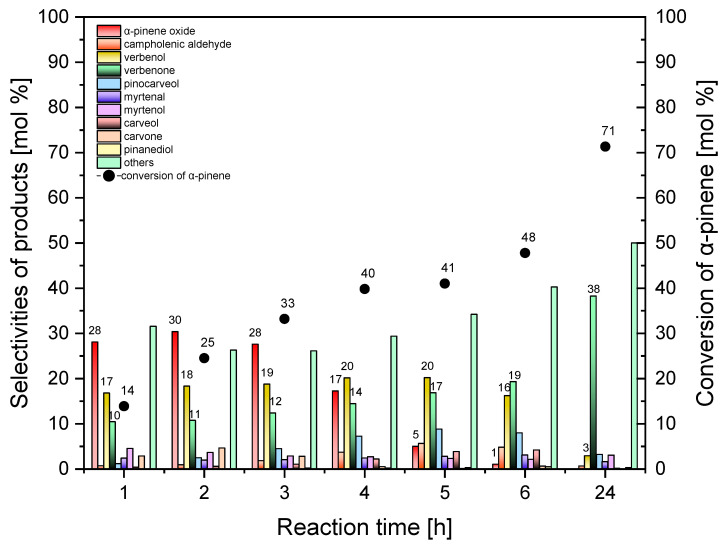
Influence of the reaction time on the conversion of α-pinene and selectivities of products in the oxidation of α-pinene over Ti-SBA-15_standard (temperature, 120 °C; catalyst content, 0.05 wt%). “Others” refers to the sum of the selectivities of by-products of the α-pinene oxidation process that were formed in smaller amounts.

Figure 10 shows the results of the influence of reaction time on the main function, describing the process of α-pinene oxidation over the standard Ti-SBA-15 catalyst.

Figure 10 shows that, with the prolongation in the reaction time 1 to 24 h, the selectivity of the transformation to the epoxy compound decreases from 28 mol% to 0 mol%; the highest value of this function of the process was obtained for the reaction time of 2 h, and it amounted to 30 mol%. The selectivity of transformation to verbenol remains at the level of 16–20 mol% during the reaction time in the range of 1–6 h, while for the reaction time of 24 h the selectivity of transformation to this compound amounts to only 3 mol%. As the reaction time increases from 1 to 24 h, the selectivity of transformation to verbenone increases from 10 mol% to 38 mol%. Particularly noteworthy is the very high selectivity of the transformation to this compound achieved for the reaction time of 24 h. With the increase in the reaction time, the conversion of α-pinene also increases from 14 mol% to 71 mol%. The reaction time of 3 h can be considered as the most favorable reaction time for oxidation which was performed with the standard SBA-15 catalyst, considering mainly the selectivities of the transformation to the three main products: alpha-pinene oxide (28 mol%), verbenol (19 mol%), verbenone (12 mol%), and in second place the conversion of α-pinene (33 mol%).

**Figure 11:**
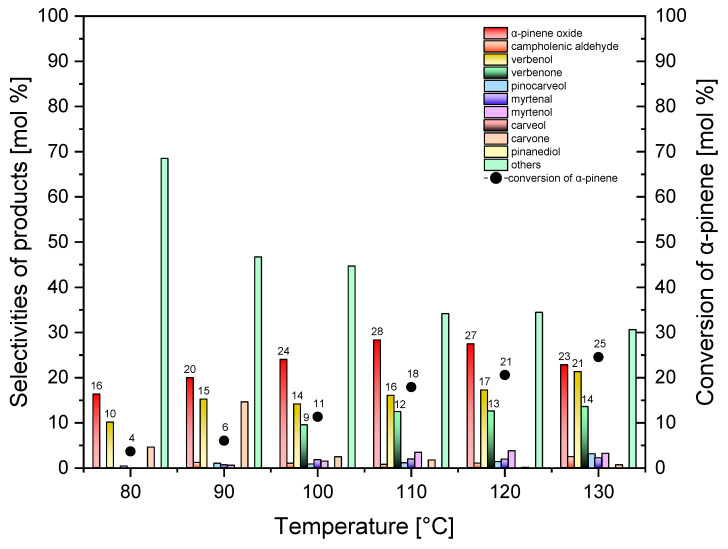
Influence of temperature on the conversion of α-pinene and selectivities of products in the oxidation of α-pinene over the Ti-SBA-15_orange peel catalyst (catalyst content, 0.5 wt%; reaction time, 1 h). “Others” refers to the sum of the selectivities of by-products of the α-pinene oxidation process that were formed in smaller amounts.

**Figure 12:**
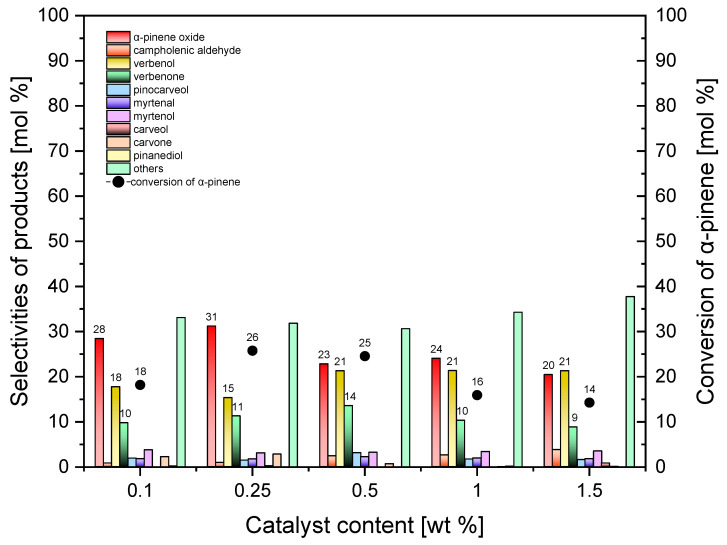
Influence of the catalyst content on the conversion of α-pinene and selectivities of products in the oxidation of α-pinene over Ti-SBA-15_orange peel catalysts (temperature, 130 °C; reaction time, 1 h). “Others” refers to the sum of the selectivities of by-products of the α-pinene oxidation process that were formed in smaller amounts.

**Figure 13:**
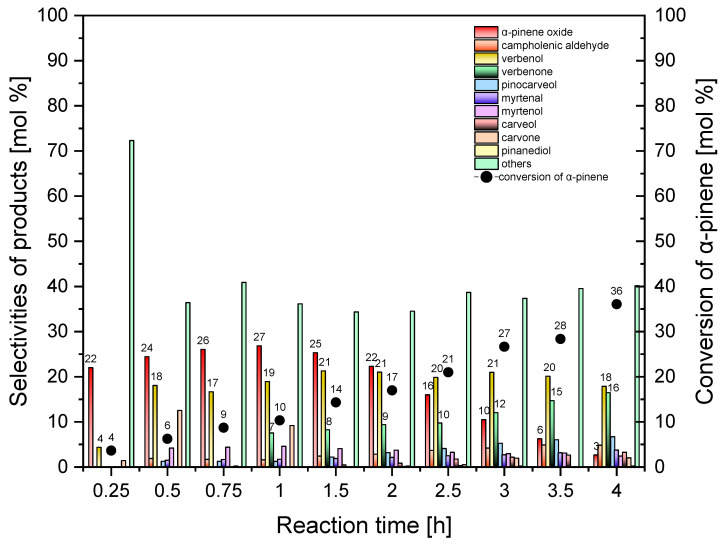
Influence of the reaction time on the conversion of α-pinene and selectivities of products in the oxidation of α-pinene over Ti-SBA-15_orange peel catalysts (temperature, 130 °C; catalyst content, 0.25 wt%). “Others” refers to the sum of the selectivities of by-products of the α-pinene oxidation process that were formed in smaller amounts.

Figure 11, Figure 12 and Figure 13 show the results of studies on the catalytic activity of Ti-SBA-15_orange peel catalyst. The studies were performed in the temperature range of 80–130 °C, for the catalyst content of 0.1–1.5 wt%, and for the reaction time in the range of 0.25–4 h. Figure 11 shows the effect of temperature on the selectivities of transformation to the appropriate products and on the conversion of α-pinene.

Figure 11 shows that, as the temperature of the oxidation process increases from 80 °C to 110 °C, the selectivity of the transformation to α-pinene oxide rises from 16 mol% to 27 mol% and then slightly decreases to 23 mol% at the temperature of 120 °C. The comparison shows that the values of selectivity of the transformation to α-pinene oxide obtained during the tests of the influence of temperature are higher for the Ti-SBA-15_orange peel catalyst than those obtained earlier for the standard Ti-SBA-15 catalyst; the comparison of the most favorable conditions and values of the main functions describing the course of the oxidation process for both tested catalysts is presented in Table 2.

The greatest differences are observed at the lowest temperatures tested, i.e., 80 to 100 °C, and these differences range from 11 mol% to 6 mol%. The increase in the selectivity of the transformation to the epoxy compound at lower temperatures of the process is very beneficial, taking into account the economics of the process carrying out.

The selectivity of the transformation to verbenol changes from 10 to 21 mol% within the studied temperature range. The obtained selectivity values of this compound are slightly higher than in the case of tests conducted for the standard Ti-SBA-15 catalyst, especially for the two lowest temperatures of 80 and 90 °C (a difference of about 3 and 5 mol%). A higher value of about 4% mol was also obtained for the highest tested temperature, i.e., 130 °C.

At the temperatures of 80 °C and 90 °C, the selectivity of the transformation to verbenone is 0 mol%, while in the temperature range of 100–130 °C, the selectivity of this compound ranged from 9 mol% to 14 mol%. The comparison with the standard Ti-SBA-15 catalyst shows that there is a difference between these catalysts in terms of directing the reaction towards verbenone. If we assume that verbenone is mainly formed from verbenol, then, at the two lowest temperatures, high stability of the verbenol molecule is observed, and only from 100 °C does partial oxidation of verbenol to verbenone occurs. For the two highest temperatures tested (120 and 130 °C), a slightly higher selectivity of the transformation to verbenone is observed.

Figure 11 also shows that the conversion of alpha-pinene rises from 4 mol% to 25 mol% during the increasing in the temperature of the oxidation process. The comparison of both tested catalysts in terms of the obtained conversions indicates that higher conversion values were obtained over the Ti-SBA-15_orange peel catalysts. The highest difference was noted for the temperature of 130 °C and amounted to 11 mol%.

Based on the conducted at this stage of studies, it was established that the most beneficial reaction temperature is the temperature of 130 °C (taking into account the selectivities of the three main products and the conversion of the organic raw material).

The results of the studies on the effect of catalyst (Ti-SBA-15_orange peels) content are presented in Figure 12. The oxidation was conducted at the temperature of 130 °C, which was identified as the most beneficial in the first stage of studies. The range of catalyst content was 0.1–1.5 wt%, and the reaction time amounted to 1 h.

Figure 12 shows that the selectivity of the transformation to α-pinene epoxide raises from 28 mol% to 31 mol% (for the catalyst content 0.25 wt%) and decreases to 20 mol%. For lower than 0.1 wt% content of Ti-SBA-15_orange peel catalyst, no oxidation reaction was observed. Different results were observed for the standard Ti-SBA-15 catalyst because the oxidation reaction occurred for this catalyst, at its content of 0.025 and 0.05 wt% (Table 2), which indicates higher activity of this catalyst, even though the reaction was carried out at lower temperature (120 °C). The comparison also shows that, for the two catalyst contents of 1 and 1.5 wt%, higher selectivity of the transformation to α-pinene oxide (by 5 and 9 mol%, respectively) was obtained over the Ti-SBA-15_orange_peel catalysts. This may be due to, among others, the higher temperature of the oxidation process on this catalyst, but also probably to the higher stability of this compound in the larger pores of the Ti-SBA-15_orange peel catalysts.

The selectivity of the transformation to verbenol changes from 18 mol% to 21 mol% in the whole range of the tested catalyst contents. The comparison of the results obtained for the Ti-SBA-15_orange peel catalysts with the results for the standard Ti-SBA-15 catalyst shows that, for catalyst contents in the range of 0.5 to 1.5, the obtained selectivities of transformation to verbenol were higher for the Ti-SBA-15_orange peel catalysts (by 4 to 7 mol%).

The selectivity of the transformation to the last main reaction product (verbenone) ranges from 9 mol% to 14 mol% over the entire range of catalyst contents. The comparison of the obtained results with those obtained for the standard Ti-SBA-15 catalyst showed a very high similarity in the selectivities of the transformation to verbenone.

The conversion of α-pinene over the Ti-SBA-15_orange peel catalysts varied from 14 to 26 mol%, with the highest values of this function obtained for the catalyst contents of 0.25 and 0.5 wt%. Comparison of the two catalysts tested shows that similar values of α-pinene conversion were obtained, but with the exception of the above-mentioned two catalyst contents, for which the conversion on the Ti-SBA-15_orange peel catalysts was higher than over the standard Ti-SBA-15 catalyst.

After the analysis of the obtained results for Ti-SBA-15_orange peels, the catalyst content of 0.25 wt% was taken as the most beneficial (taking into account the selectivities of the main products and the conversion of the organic raw material).

In the last stage of the catalytic studies, the aim was to investigate how reaction time affects the selectivity of the transformation to the appropriate products and the conversion of α-pinene in the presence of the mesoporous material, Ti-SBA-15_orange peel, as the catalyst. The oxidation of α-pinene was conducted in the range of reaction times from 0.25 to 4 h at the temperature of 130 °C, with the catalyst content amounting to 0.25 wt%. The obtained results are presented in Figure 13.

During the tests of the Ti-SBA-15_orange peel catalysts, the reaction of oxidation was observed for reaction times of 15, 30, and 45 min. For the standard Ti-SBA-15 catalyst, no reaction was observed for such short reaction times, which indicates the higher activity of the Ti-SBA-15_orange peel catalysts compared to the standard Ti-SBA-15 catalysts (Table 2). At the same time, due to the 0 mol% selectivity of the transformation to α-pinene oxide observed for reaction times above 4 h, the time-effect tests for longer reaction times than 4 h were abandoned.

During prolongation of the reaction time from 0.25 h to 1 h, the selectivity of the transformation to α-pinene oxide rose from 22 mol% to 27 mol%; next was at the level of 22–25 mol% for the reaction time from 1.5 to 2 h, and for the reaction time above 2 h, it decreased slowly to the value of 3 mol% (reaction time of 4 h), as shown in Figure 13. The comparison of the results in the range of reaction times from 1 to 4 h for both tested catalysts showed that, for the Ti-SBA-15_orange peel catalysts, significantly lower values of the selectivity of the transformation to α-pinene oxide were obtained (for the reaction time of 4 h, even six times the lower value). This indicates a high instability of the formed epoxy compound under the tested conditions, which most probably underwent transformation to oligomeric products immediately after its formation, as indicated by the higher values of selectivity to other reaction products (total selectivity marked as “others” in Figure 13) on the Ti-SBA-15_orange peel catalysts—even by 11 mol% for the reaction time of 4 h.

For the reaction time of 0.25 h, the selectivity of the transformation to verbenol amounted to 4 mol%; next it rose to 18–20 mol%. The analysis shows that similar results were obtained for the standard Ti-SBA-15 catalyst.

For the reaction time of 15 min to 60 min, the formation of verbenone was not observed. This compound is detected first in the post-reaction mixture for the reaction time of 90 min (selectivity of 8 mol%), and the value of this function rose to 16 mol% during prolongation of the reaction time to 4 h. The comparison of both tested catalysts showed that verbenone was already formed on the standard Ti-SBA-15 catalyst for the reaction time of 1 h. For the remaining reaction times in the range of 2–4 h, the obtained transformation selectivities for this compound were similar for both catalysts.

The conversion of α-pinene rose from 4 mol% to 36 mol% in the studied range of the reaction time from 15 min to 240 min (4 h). The comparison of the results obtained for both tested catalysts for reaction times of 1 to 4 h showed that higher values of α-pinene conversion were obtained for the standard Ti-SBA-15 catalyst (values higher by up to 8 mol%).

It was established based on the conducted studies that the most favorable reaction time in the case of the Ti-SBA-15_orange peel catalysts is the reaction time of 1.5 h. For this reaction time, the selectivities of the main products were as follows: α-pinene oxide 25 mol%, verbenol 21 mol%, and verbenone 8 mol%, and the conversion of α-pinene was 14 mol%.

In the case of the standard Ti-SBA-15 (Figure 10), which is characterized by narrower pores, the onset of the reaction is observed only after one hour, and as the reaction time increases, a gradual rise in α-pinene conversion takes place. However, the rate of this increase may be limited by the diffusion of reactants and products within the mesoporous structure—particularly in the later stages of the reaction, where slower diffusion begins to play a more significant role. This can lead to a saturation effect, where, despite the presence of catalytically active sites, further conversion is limited by difficulties in transporting molecules to and from those sites.

In contrast, in the presence of Ti-SBA-15_orange peels (Figure 13), the reaction is observed as early as after 15 min. Despite its lower specific surface area, a more stable and systematic increase in conversion over time is observed, which can be attributed to the presence of wider pores. These pores facilitate better diffusion of α-pinene and the reaction products, minimizing transport limitations. In this case, the dominant mechanism controlling the reaction rate is the chemical reaction kinetics rather than diffusion resistance. As a result, conversion increases more effectively at longer reaction times, and the active centers are more efficiently utilized.

Importantly, the product selectivity profile does not exhibit significant fluctuations over time when using the catalyst with wider pores, which further suggests that the system is governed more by the chemical properties of the catalyst than by physical limitations related to mass transport.

The experimental results presented in Figure 10 and Figure 13 clearly illustrate the impact of pore size on the reaction mechanism during α-pinene oxidation. For the standard Ti-SBA-15 catalyst, with narrower pores, the reaction shows signs of diffusion limitations; the conversion increases slowly over time and may reach a plateau due to restricted mass transport. In contrast, the Ti-SBA-15_orange peel catalysts, with its wider pores, enables more efficient diffusion of α-pinene and its oxidation products, resulting in a more linear and sustained increase in conversion. This confirms that, in catalysts with narrower pores, the reaction is predominantly diffusion-controlled, whereas in those with broader pores, the kinetics of the chemical reaction itself governs the overall performance. These observations further emphasize the advantages of wider-pore mesostructures in catalytic processes involving bulky organic molecules.

**Figure 14:**
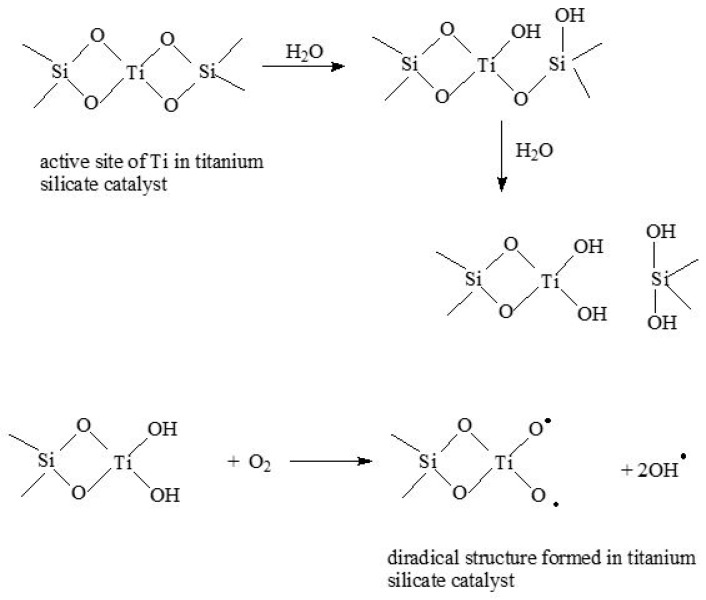
Formation of diradical structure in titanium silicate catalyst.

**Figure 15:**
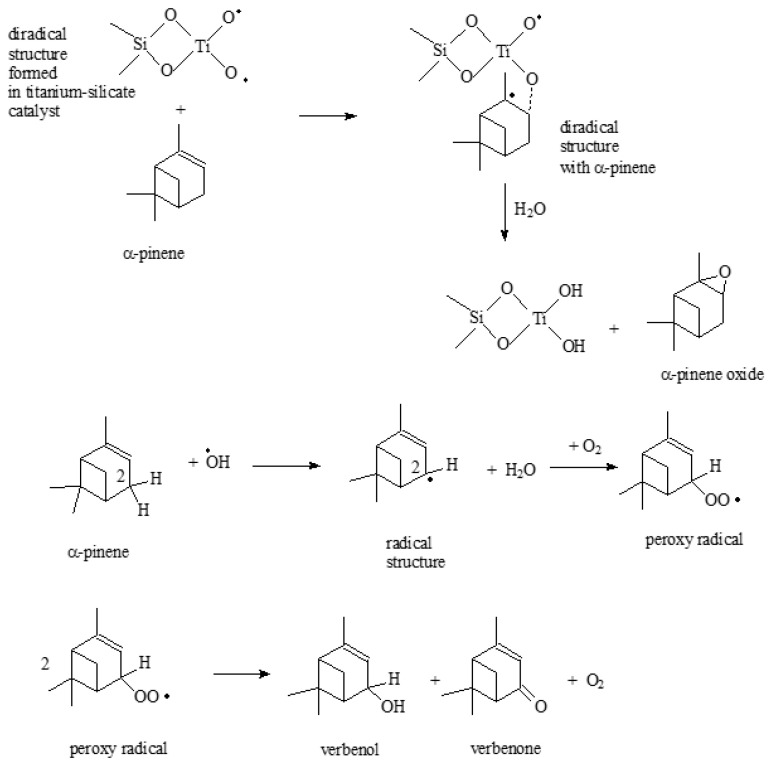
Possible ways of creating α-pinene oxide, verbenol, and verbenone.

As it results from the above-mentioned results of catalytic studies for the standard Ti-SBA-15 and Ti-SBA-15_orange peel catalysts, the oxidation process of a-pinene is a very complex process, and its mechanism of occurrence is very difficult to explain in a simple way. Based on our earlier publication and publications of other authors [27,50,55,56,57], the following routes for the formation of the main products of a-pinene oxidation, i.e., a-pinene oxide, verbenol, and verbenone, can be proposed (Figure 14 and Figure 15).

Figure 14 shows the way of formation to a diradical structure in a titanium silicate catalyst. This structure is formed in both the standard Ti-SBA-15 catalyst and the Ti-SBA-15_orange peel catalyst. The process of creating this structure starts with the hydration of the active center of titanium, as a result of which two -OH groups appear at the Ti atom, which is bound in the silica structure. In the next step, a reaction with oxygen takes place, as a result of which a diradical structure is formed, which takes part in the next steps, in which oxygen derivatives of a-pinene are formed.

Figure 15 shows a probable way of α-pinene oxide formation, during which a diradical structure is formed. It is formed as a result of an interaction of the diradical structure, produced in the titanium silicate catalyst, with the participation of the active center in the form of Ti tetrahedrally bonded in the silica structure, with the α-pinene molecule. Later, hydrolysis of this diradical structure takes place, and α-pinene oxide is formed, and two hydroxyl groups are recreated at the Ti atom. In the formation of verbenol and verbenone hydroxyl radicals play a key role (Figure 15). These radicals interact with the a-pinene molecule, as a result of which the H atom located in position 2 in the α-pinene molecule is removed. As a result of the removal of a proton, a radical structure is formed, and a water molecule is formed. This radical structure then reacts with the oxygen molecule, as a result of which a peroxy radical is formed. In the next step, a verbenol and verbenone molecule is formed from two peroxiradicals.

## 3. Materials and Methods

### 3.1. Raw Materials

In the tested process, the following reagents were used for the oxidation carried out with the use of two mesoporous, titanium-silicate Ti-SBA-15 catalysts: α-pinene (98%, Sigma Aldrich, Poznań, Poland) and oxygen (99.99%, Messer, Szczecin, Poland). Moreover, the following compounds were used as the standards for chromatographic analyses: α-pinene oxide (97%, Sigma Aldrich, Poznań, Poland), verbenol (95%, Sigma Aldrich, Poznań, Poland), verbenone (93%, Sigma Aldrich, Poznań, Poland), trans-pinocarveol (96%, Sigma Aldrich, Poznań, Poland), myrtenal (98%, Sigma Aldrich, Poznań, Poland), myrtenol (95%, Sigma Aldrich, Poznań, Poland), carveol (95%, Sigma Aldrich, Poznań, Poland), carvone (98%, Sigma Aldrich, Poznań, Poland), and pinanediol (99%, Sigma Aldrich, Poznań, Poland). Campholenic aldehyde was identified by the GC-MS method.

### 3.2. Synthesis of the Ti-SBA-15 Materials

#### 3.2.1. Synthesis of the Standard Ti-SBA-15 Catalyst

The standard Ti-SBA-15 catalyst was prepared by the hydrothermal method described by Berube et al. [32]. The obtained catalyst contained 0.5 wt% titanium. The method of the synthesis was as follows: 18 g of template (a biodegradable, triblock copolymer of ethylene oxide and propylene oxide, Pluronik P123) was dissolved at 35 °C in a mixture of 376 g of water and 10.5 HCl (37% aqueous solution). Then, a mixture of 39.0 g of TEOS and TiPOT (1.80 g for Si:Ti = 30:1) was quickly added to this solution. The resulting mixture was vigorously stirred and kept at 35 °C for 24 h, after which the resulting crystallization gel was left without stirring for another 24 h at 35 °C. The resulting catalyst was filtered, washed with deionized water, and then dried at 100 °C for 24 h. Finally, the resulting material was calcined for 5 h at 550 °C. In this way, the standard Ti-SBA-15 material was obtained in the form of a white powder.

#### 3.2.2. Synthesis of the Ti-SBA-15_Orange Peel Material

The Ti-SBA-15_orange peel catalyst, with the titanium content of 0.5 wt%, was obtained based on the method described by Berube et al. [32].

This catalyst was prepared using orange peel waste (used as the additional template—co-template), which were dried, ground in a mill with boiling water, and hot-filtered, followed by drying in the oven at 100 °C. The synthesis of Ti-SBA-15_orange peels can be described as follows: To the glass reactor equipped with the reflux condenser and mechanical stirrer, which was placed in the oil bath at 35 °C, the following raw materials were added sequentially: 8.09 g of Pluronic P123, 168.88 g of deionized water, and 4.72 cm^3^ of HCl. The content of the reactor was stirred until a clear solution was obtained, after which the mixture of tetraethyl orthosilicate (17.52 g) and tetraisopropyl orthotitanate (0.81 g) was added along with 13.33 g of the previously prepared orange peel waste (the mass ratio of liquid to peel waste was 15:1). The content of the reactor was stirred for 24 h, and then stirring was stopped, and the content was left for another 24 h. After this time, the precipitate was filtered, washed with deionized water, dried at 100 °C for 24 h, and calcined at 550 °C for 5 h.

Table 3 shows the amounts of raw materials used for the synthesis of Ti-SBA-15 catalysts.

### 3.3. Characteristics of the Ti-SBA-15 Catalysts

The textural characteristics of the materials were evaluated using nitrogen adsorption–desorption measurements conducted at −196 °C, employing a QUADRASORB evo™ Gas Sorption Surface Area and Pore Size Analyzer (Anton Paar, St Albans, UK; previously Quantachrome Instruments, Boynton Beach, USA, 2014). Prior to analysis, all samples underwent degassing at 250 °C for a minimum of 20 h to ensure the complete removal of adsorbed species. The specific surface area was determined using the Brunauer–Emmett–Teller (S_BET_) model. The pore size distribution was calculated using the BJH method. The total pore volume (V_tot_) was determined from the nitrogen adsorption volume at a relative pressure near 1.

The structural characterization of the catalysts was performed using X-ray diffraction (XRD) analysis on a PANalytical Empyrean diffractometer (Almelo, The Netherlands, 2012), employing Cu Kα radiation as the source. Data were collected in the 2θ range of 0.1–3° with a step size of 0.013° for low-angle analysis and in the 2θ range of 10–35° for wide-angle measurements. The crystallite size of TiO_2_ was estimated using the Scherrer equation.

The morphological analysis of the samples was carried out using an SU8020 Ultra-High Resolution Field Emission Scanning Electron Microscope (Hitachi Ltd., Ibaraki, Japan, 2012). EDX analyses were performed with a Thermo Fisher Scanning Electron Microscope Apreo 2S (Thermo Fisher Scientific, Waltham, MA, USA) in high vacuum.

### 3.4. Oxidation of α-Pinene

The apparatus used for the oxidation of α-pinene consisted of the following components: 1—oxygen cylinder, 2—oxygen flow regulator, 3—glass reactor with the capacity of 25 cm^3^, 4—glass bubbler for oxygen supply, 5—reflux condenser, 6—thermometer, and 7—magnetic stirrer with heating function. The scheme of the apparatus used to the oxidation of α-pinene was presented in our previous publication [50].

The appropriate amount of α-pinene (about 10 g for testing the effect of temperature and content of the catalyst and about 20 g for testing the effect of the reaction time) was introduced into the reactor. Then, the catalyst was added in the amount ranging from 0.025 to 1.5 wt%, and oxygen was fed into the reactor at the rate of 40 mL/min. The reaction was carried out between 80 and 130 °C for 0.25 to 24 h at the stirring rate of 500 rpm. The reaction mixture was collected into a 1 cm^3^ Eppendorf tube and then placed in a centrifuge to separate the catalyst from it. A 0.250 mL sample of the post-reaction mixture was then taken, and 0.750 mL of acetone was added to it. The sample prepared in this way was subjected to the chromatographic analyses.

### 3.5. Identification of the Products of Oxidation by the Gas Chromatography Method

A Thermo FOCUS instrument (Anchem, Warszawa, Poland, 2009) with an FID detector and a ThermoQuest instrument with a mass detector were used to perform qualitative and quantitative analyses of the previously prepared samples of the post-reaction mixture. A detailed description of the methods of analyses is presented in our earlier publication [58].

## 4. Conclusions

The conducted studies allowed production of an active, mesoporous Ti-SBA-15 catalyst using orange peel waste as co-templates (bio-templates) in the synthesis gel. Comparison of the activity of the Ti-SBA-15 catalyst obtained with the use of orange peels (Ti-SBA-15_orange peels) with the standard Ti-SBA-15 catalyst indicates that this catalyst, with appropriately selected conditions of the α-pinene oxidation process, may be more active than its counterparts synthesized in the standard manner. This was particularly noticeable in the case of studies of the effect of temperature in the range of 80–100 °C (the remaining test conditions were the same for both catalysts), during which the higher selectivity of the transformation to α-pinene oxide was observed for this catalyst. Moreover, the results of the temperature influence tests showed that conducting the oxidation process in the range of 80–90 °C can inhibit the oxidation of verbenol to verbenone (verbenol molecules are stable in the conditions in which the oxidation process is conducted). This phenomenon was not observed previously on the standard Ti-SBA-15 catalyst. On the other hand, at temperatures of 120–130 °C, slightly higher selectivity of the transformation to verbenone is observed for the Ti-SBA-15_orange peel catalyst. Also, the conversions of the organic substrate obtained in the presence of the Ti-SBA-15_orange peel catalyst were higher than during the tests conducted for the standard Ti-SBA-15 catalyst, while the difference in the conversion values for both catalysts increased with the increase in the process temperature. At the same time, it should be noted that the total selectivity of the transformation to other products (referred to in the figures as the “others” transformation selectivity) was similar for both catalysts, which indicates that the larger pores of the Ti-SBA-15_orange peel catalyst did not promote the significant increase in side reactions, mainly reactions related to the oligomerization of the main reaction products and the organic substrate.

Studies on the effect of the content of the catalyst showed that oxidation using the Ti-SBA-15_orange peel catalyst requires the use of larger amounts of this catalyst compared to the standard Ti-SBA-15 catalyst, even if the oxidation studies are carried out at higher temperatures (in our studies conducted on the Ti-SBA-15_orange peels catalyst, the temperature was 10 °C higher than in studies using the standard Ti-SBA-15 catalyst). At very low contents of this catalyst (0.025 and 0.05 wt%), the reaction did not occur. However, for the highest tested catalyst contents (1 and 1.5 wt%), a higher selectivity of transformation to α-pinene oxide and verbenol was observed, while maintaining similar selectivities of transformation to verbenone and similar conversions of the organic raw material. The results obtained during the studies of the effect of the content of catalyst indicate that the larger pores of the Ti-SBA-15_orange peel catalyst facilitate the diffusion of α-pinene molecules into their interior and reaching the active centers where the reaction takes place, and at the same time they provide stabilization to the formed products, which prevents their easy transformation into oligomeric products, because the products named as “others” are formed at the highest catalyst contents with lower selectivity on the Ti-SBA-15_orange peel catalyst.

The catalytic activity of the Ti-SBA-15_orange peel catalyst was already observed for such short reaction times as the following: 15 min, 30 min, and 45 min. This indicates a higher activity of this catalyst; because for the standard Ti-SBA-15 catalyst, the reaction was not observed for such short reaction times. At the same time, for this catalyst, the formation of α-pinene oxide was not observed for reaction times above 4 h. This compound probably underwent further changes in the pores—mainly oligomerization reactions. Therefore, there is no stabilizing effect of the pores on this product (an effect increasing its durability in the pores); on the contrary, oligomerization processes are intensified, which can be seen from the increasing selectivity values of the products named as “others”. It can be assumed that this is related to the larger pore size of the Ti-SBA-15_orange peel catalyst and therefore to the accumulation of both products and unreacted substrate in them, which affects the subsequent reactions involving α-pinene oxide. However, the stabilizing effect of the pores is visible in the case of another compound (verbenol) and for short reaction times, namely 15 and 30 min. For these reaction times, verbenone, which is formed by oxidation of verbenol, is not detected in the post-reaction mixture. On this basis, it can be assumed that the catalyst pores affect the stability of this product here.

In summary, it can be said that the catalyst obtained with the participation of orange peels (bio-templates) is a very promising and active catalytic material in oxidation processes. Its advantage is that for its synthesis we use renewable waste from the food industry, which is difficult to manage—orange peels. Further research on this catalyst should go towards increasing the share of peels in the composition of the crystallization gel, so as to partially replace the standard template used in the synthesis of this catalyst.

The Ti-SBA-15 catalyst, obtained in this work with the participation of orange peels as co-templates, may find numerous applications in the organic industry in the future. The main applications of this catalyst may be related to the processes of oxidation or isomerization of unsaturated compounds, including terpenes of natural origin; as a result of the transformations of these compounds are obtained very valuable biologically active compounds for medicine and cosmetics as well as components of fragrance and flavor compositions for the perfume and food industry. However, this requires the development of a method of catalyst synthesis on a larger scale. It should be emphasized that the method of synthesis of this catalyst is easier than the method of synthesis of synthetic zeolites, e.g., catalyst TS-1 (the catalyst that has also found application in industry, e.g., in the process of phenol hydroxylation or propylene oxidation), and proceeds under milder conditions (low temperature and short crystallization time). The only problem to be solved would be the delivery of orange peel waste, which are used as co-templates. The solution would be to locate plants producing such catalysts near plants producing orange juice or to deliver frozen orange peels to the plant producing the catalyst.

## Figures and Tables

**Table 1:** Textural properties of standard Ti-SBA-15 and Ti-SBA-15_orange peels and content of Ti established by the EDX method.

Catalytic Material	S_BET_ [m^2^/g]	V_tot_ [cm^3^/g]	V_mic_ [cm^3^/g]	Ti [wt%]
Ti-SBA-15	771	0.631	0.121	0.5
Ti-SBA-15_orange peels	397	0.551	0.011	0.5

**Table 2:** Oxidation of a-pinene on standard Ti-SBA-15 and Ti-SBA-15_orange peel catalysts: comparison of the most favorable conditions and values of the main functions describing the course of the oxidation process.

Standard Ti-SBA-15							
Studied Parameter	Temperature (°C)	Catalyst Content (wt%)	Reaction Time (h)	* C_α-pinene_ (mol%)	* S_α-pinene oxide_ (mol%)	* S_verbenol_ (mol%)	* S_verbenone_ (mol%)
**Temperature**	80	0.5	1	3	5	7	14
	90			5	12	10	18
	100			9	17	14	12
	110			12	24	17	11
	**120**			18	25	17	11
	130			13	19	17	10
**Catalyst** **content**	120	0.025	1	22	32	17	11
		**0.05**		24	29	18	10
		0.1		21	28	17	10
		0.25		13	29	18	10
		0.5		18	25	17	11
		1		15	19	17	10
		1.5		14	12	14	9
**Reaction** **time**	120	0.05	1	14	28	17	10
			2	25	30	18	11
			**3**	33	28	19	12
			4	40	17	20	14
			5	41	5	20	17
			6	48	1	16	19
			24	71	0	3	38
**Ti-SBA-15_orange peels**							
**Temperature**	80	0.5	1	4	16	10	0
	90			6	20	15	0
	100			11	24	14	9
	110			18	28	16	12
	120			21	27	17	13
	**130**			25	23	21	14
**Catalyst** **content**	130	0.1	1	18	28	18	10
		**0.25**		26	31	15	11
		0.5		25	23	21	14
		1		16	24	21	10
		1.5		14	20	21	9
**Reaction time**	130	0.25	0.25	4	22	4	0
			0.5	6	24	18	0
			0.75	9	26	17	0
			1	10	27	19	7
			**1.5**	14	25	21	8
			2	17	22	21	9
			2.5	21	16	20	10
			3	27	10	21	12
			3.5	28	6	20	15
			4	36	3	18	16

* C_α-pinene_—conversion of α-pinene; S_α-pinene oxide_—selectivity of α-pinene oxide; S_verbenol_—selectivity of verbenol; S_verbenone_—selectivity of verbenone.

**Table 3:** Amounts of raw materials used for the syntheses of Ti-SBA-15 catalysts.

	Pluronic P123 [g]	TEOS [g]	TiPOT [g]	HCl [mL]	H_2_O [g]	Orange Peel Waste [g]
Ti-SBA-15	18.04	37.20	1.80	10.51	376.04	-
Ti-SBA-15_orange peels	8.09	17.52	0.81	4.72	168.88	13.33

## Data Availability

The original contributions presented in this study are included in the article. Further inquiries can be directed to the corresponding author.
